# Effects of plyometric and isometric training on muscle and tendon stiffness in vivo

**DOI:** 10.14814/phy2.13374

**Published:** 2017-08-11

**Authors:** Keitaro Kubo, Tomonobu Ishigaki, Toshihiro Ikebukuro

**Affiliations:** ^1^ Department of Life Science University of Tokyo Meguro Tokyo Japan

**Keywords:** Fascicle, plantar flexion, tendon structures

## Abstract

The purpose of this study was to compare the effects of plyometric and isometric training on tendon properties during ramp and ballistic contractions and muscle stiffness under passive and active conditions. Eleven subjects completed 12 weeks (3 days/week) of a unilateral training program for the plantar flexors. They performed plyometric training on one side (PLY) and isometric training on the other side (ISO). Active muscle stiffness in the medial gastrocnemius muscle was calculated according to changes in estimated muscle force and fascicle length during fast stretching after submaximal isometric contractions. Passive muscle stiffness was also calculated from estimated passive muscle force and fascicle length during slow passive stretching. Stiffness and hysteresis of tendon structures were measured using ultrasonography during ramp and ballistic contractions. Passive muscle stiffness and tendon hysteresis did not change for PLY or ISO. Active muscle stiffness significantly increased for PLY, but not for ISO. Tendon stiffness during ramp and ballistic contractions increased significantly for ISO, but not for PLY. In addition, tendon elongation values at force production levels beyond 100 N during ballistic contractions increased for PLY. These results suggest that plyometric training (but not isometric training) enhances the extensibility of tendon structures during ballistic contractions and active muscle stiffness during fast stretching, and these changes may be related to improved performances during stretch‐shortening cycle exercises.

## Introduction

Plyometric training is generally expected to enhance performances during stretch‐shortening cycle exercises, for example, jumping and sprinting. Furthermore, previous studies demonstrated that the mechanical properties of the muscle‐tendon complex change after plyometric training (e.g., Kubo et al. 2007b; Spurrs et al. 2003). For example, Kubo et al. (2007b) reported that jump performances and ankle joint stiffness significantly increased after 12 weeks of plyometric training, but not after weight training. Spurrs et al. (2003) demonstrated that musculotendinous stiffness in the ankle joint measured by the oscillation technique significantly increased after 6 weeks of plyometric training, and running economy and 3 km running performance simultaneously improved. In these studies, the methodologies (e.g., the oscillation technique) used were not able to evaluate the mechanical properties of muscles and tendons separately.

In the last two decades, several studies have used ultrasonography to investigate the effects of various training protocols on human tendon properties (e.g., Kubo et al. 2007b). To date, isometric training has been shown to markedly increase tendon stiffness (Burgess et al. 2007; Kubo et al. 2012). On the other hand, there are conflicting findings on the effects of plyometric training on tendon properties. Kubo et al. (2007b) and Foure et al. (2009) reported no significant change in tendon stiffness after plyometric training, whereas other studies found significant increases (Burgess et al. 2007; Foure et al. 2010b; Wu et al. 2010). Furthermore, only one study showed a change in tendon hysteresis after plyometric training (Foure et al. 2010b), although tendon hysteresis affected performance during stretch‐shortening cycle exercises (Kubo et al. 2005; Wang et al. 2012). In these studies (Burgess et al. 2007; Kubo et al. 2007b; Foure et al. 2009, 2010b; Wu et al. 2010), stiffness and hysteresis of tendons were investigated during ramp isometric contractions, and the strain rates of tendons were markedly different from those during running and jumping. Therefore, tendon properties need to be investigated during a high elongation rate of stretching (i.e., ballistic contractions) in order to clarify plyometric training induced‐changes in tendon properties and performance.

Previous studies demonstrated the mechanical properties of muscles from passive torque (force) and joint angles (or fascicle length) during slow passive stretching (Muraoka et al. 2002; Hoang et al. 2007). Regarding the mechanical properties of muscle under active conditions, Foure et al. (2010a) adapted the alpha method (developed by Morgan (1977)) during submaximal voluntary contractions, and reported that the passive component (i.e., tendon) of stiffness increased, while the active part component (i.e., muscle) of stiffness decreased after 14 weeks of plyometric training (Foure et al. 2011). However, a stiffer muscle is more desirable under active conditions to store more elastic energy within the tendons during stretch‐shortening cycle exercises. We recently reported that muscle stiffness could be assessed under active conditions in vivo through observations of the lengthening of fascicles during fast stretching (Kubo 2014). Furthermore, active muscle stiffness and tendon stiffness using ultrasonography were not related to active (i.e., muscle) or passive (i.e., tendon) stiffness in the series elastic component by the alpha method (Kubo 2014). These findings indicated that we need to investigate the effects of plyometric training on active muscle stiffness assessed by directly measured length changes in fascicles during fast stretching.

In this study, we aimed to compare the effects of plyometric training and isometric training on tendon properties (stiffness and hysteresis) during ramp and ballistic contractions and muscle stiffness under passive and active conditions. The results obtained were expected to provide an insight into the mechanisms underlying increases in joint stiffness and improved performance during stretch‐shortening cycle exercises after plyometric training (e.g., Kubo et al. 2007b).

## Methods

### Subjects

Eleven healthy males (age: 22.5 ± 3.2 years, height: 172.2 ± 2.5 cm, body mass: 60.9 ± 6.5 kg, mean ± SD) voluntarily participated in this study. They did not have experience of regular exercise training. They were fully informed of the procedures to be utilized as well as the purpose of this study. Written informed consent was obtained from all subjects. This study confirmed to the *Declaration of Helsinki* and was approved by the office of the Department of Sports Sciences, The University of Tokyo, and complied with their requirements for human experimentation.

### New and noteworthy points in this study

This is the first study to examine the effect of plyometric and isometric training on muscle stiffness under active condition and tendon properties during ballistic contractions. In the previous studies (e.g., Kubo et al. 2002), the muscle properties under passive conditions and tendon properties during ramp contractions were so far investigated. In this study, the mechanical properties of muscle and tendon structures in a near‐actual human movements (e.g., running and jumping) were obtained by these new methods.

### Training

Subjects completed training three times per week for 12 weeks. One leg performed plyometric training (PLY) and the other leg performed isometric training (ISO). In each subject, the right and left legs were allocated to the training protocols in a random manner. The measurements of a one‐repetition maximum (1RM) for PLY and a maximal voluntary contraction (MVC) for ISO were conducted every 4 weeks in order to adjust the training load. At the end of the training session, 1RM and MVC significantly increased by 37.7 ± 11.3% for PLY and 27.6 ± 4.0% for ISO, respectively (both *P* < 0.001).

Regarding PLY, subjects performed two kinds of training protocols (hopping and drop jumps) on a sledge apparatus (VR‐4100, Cybex Corp.,). The protocol for PLY was the same in our previous study (Kubo et al. 2007b). During the hopping jump, subjects initially maintained a maximal plantar‐flexed position. Subjects then exerted plantar flexion torque to maximal dorsiflexion, and rebounded to start plantar flexion until the toe finally lifted away from the footplate of this apparatus. These movements were repeated without a break. During the drop jump, the sliding table of this apparatus was moved to a height of 20 cm from the surface of the footplate of this apparatus to the sole of their foot with the assistance of an experimenter. Subjects were dropped down from a height of 20 cm. After landing on the edge of the footplate of this apparatus, they arrested the falling motion by eccentrically plantar flexing. They then started plantar flexion and took off as high as possible. These movements were repeated without a break. Subjects performed five sets of each exercise (hopping and drop jumps) with a between‐set rest interval of 30 sec, which consisted of unilateral plantar flexion at 40% of 1RM with 10 repetitions per set.

Regarding ISO, subjects performed a unilateral isometric plantar flexion exercise in the prone position. The ankle joint was set at 0 deg (neutral ankle position) with the knee joint at full extension, and the foot was securely strapped to a foot plate connected to the lever arm of a dynamometer (Vine, Tokyo, Japan). The training protocol involved ten contractions (80% of MVC) of a 15‐sec duration with a 30‐sec rest between each contraction. During contractions, subjects were encouraged to maintain the target torque displayed on an oscilloscope.

### Muscle thickness and tendon cross‐sectional area

An ultrasonic apparatus (SSD‐6500, Aloka, Japan) was used to assess the muscle thickness of the plantar flexor muscles, that is, the medial gastrocnemius muscle (MG), lateral gastrocnemius muscle (LG), and soleus muscle (SOL) using the procedure described by Kubo et al. (2014). Cross‐sectional images were obtained at proximal levels of 30% (MG and LG) and 50% (SOL) of the lower leg length. At that level, the mediolateral widths of MG and LG were evaluated over the skin surface, and the position of one‐half of this width was used as a measurement site for each muscle. The position of the greatest thickness in the lateral half of SOL was measured at the level described above. Furthermore, the mean value of MG, LG, and SOL thicknesses was adopted as the muscle size of the plantar flexors (PF). After measurements of muscle thickness, the cross‐sectional area of the Achilles tendon was also measured using an ultrasonic apparatus at the height of the lateral malleolus (Kubo et al. 2014). In our previous study (Kubo et al. 2014), the repeatability of the measurements of muscle thickness and tendon cross‐sectional area was investigated on two separate days with six young males. The coefficient of variation of muscle thickness measurement was 2.2% for MG, 2.4% for LG, and 2.8% for SOL. The coefficient of variation of tendon cross‐sectional area measurement was 3.8%.

### Passive muscle stiffness during slow stretching

The joint angle, passive torque, and fascicle length were measured to assess passive muscle stiffness (Kubo 2014; Kubo et al. 2015). Subjects lay prone on a test bench with their foot tightly secured by two straps to the footplate of a specially designed dynamometer (Applied Office, Tokyo, Japan). The ankle joint was set at 10 deg (0 deg was the neutral anatomical position; positive values for plantar flexion) with the knee joint at full extension. Subjects did not warm‐up before this measurement. While subjects maintained completely relaxed muscles, the ankle was passively moved from 10 deg to −10 deg with a constant velocity of 5 deg·sec^−1^. In this study, electromyographic activity was recorded during measurements of passive muscle stiffness. Bipolar surface electrodes (5 mm in diameter) were placed over the bellies of MG, LG, SOL, and tibialis anterior muscle with constant interelectrode distance of 25 mm. During the measurement, we confirmed the absence of myoelectric activities in the plantar flexor muscles (MG, LG, and SOL) and tibialis anterior muscle. In order to minimize thixotropic effects as preconditioning (Muraoka et al. 2002; Hoang et al. 2007), we collected data during the 6th cycle after five cycles. Passive torque (TQ) measured during slow stretching was converted to muscle force (*F*_m_) using the following equation:

$$F_{m}=k\cdot \text{TQ}\cdot {\text{MA}}^{-1}$$

where *k* represents the relative contribution of the physiological cross‐sectional area of MG within plantar flexor muscles (Fukunaga et al. 1996), and MA is the moment arm length of the triceps surae muscles at 0 deg of the ankle joint, which is estimated from the lower leg length of each subject (Grieve et al. 1978).

A real‐time ultrasonic apparatus was used to obtain a longitudinal ultrasonic image of MG at the level of 30% of the lower leg length during slow stretching. Ultrasonic images were recorded on a video tape at 30 Hz and synchronized with recordings of a clock timer for subsequent analyses. Fascicle length was defined as the distance between the insertion of the fascicle into the superficial and deep aponeuroses.

Passive torque, the joint angle, and fascicle length were continuously recorded during slow stretching. The slope of the portion of passive muscle force–fascicle length curve from 0 deg to −10 deg, was defined as passive muscle stiffness (Kubo 2014; Kubo et al. 2015). In our previous study (Kubo 2014), the repeatability of passive muscle stiffness measurements was investigated on two separate days with nine young males. The test–retest correlation coefficient (*r*) and the coefficient of variation were 0.89 and 5.6%, respectively.

### Active muscle stiffness during fast stretching

The posture of the subject and setup were similar to that for the measurement of passive muscle stiffness, as described above. After a standardized warm‐up, subjects performed two or three isometric MVC at 10 deg of the ankle angle. The highest MVC value before training was used to assess the target torque during the short range stretch experiment (both before and after training).

In order to evaluate active muscle stiffness, subjects performed the short range stretch experiment using a previously described procedure (Kubo 2014; Kubo et al. 2015). The specially designed dynamometer was programmed to apply dorsiflexion stretches from 10 deg to −10 deg. Subjects were instructed to relax as soon as ankle motion was perceived according to the procedure of Blanpied and Smidt (1992). When it is fast to relax, increase in torque during fast stretching would be abnormally lower. If these phenomena are seen, the subject was requested to perform the additional measurements. On the contrary, when it is late to relax, the measured variables (increases in torque and fascicle length) do not change. The torque and fascicle length during 60‐msec period after the onset of the stretch were analyzed because this time period was selected to avoid any potential neural effects, that is, stretch reflex (Blanpied and Smidt 1992; Kubo 2014). The range of motion was approximately 8 deg during this period. Angular velocity during the stretch reached approximately 250 deg·sec^−1^. In this study, the electromyographic activities of the plantar flexor muscles (MG, LG, and SOL) were recorded (as described above), and full‐wave rectified, and averaged over two different phases: a 60‐msec period just before the stretch (mEMGa) and a 60‐msec period after the stretch (mEMGb) (Kubo et al. 2015). Before the experiment, subjects were familiarized to the short range stretch experiment at 50% MVC. An additional measurement was conducted twice at 0% MVC before the short range stretch experiment for data correction purposes. Averaged torque during the relaxed condition was subtracted from measured torque during each of the active stretch trials (Blanpied and Smidt 1992; Kubo 2014). The short‐range stretch experiment was performed at three levels of submaximal torque in a random order (two tests at 30%, 50%, and 70% MVC) with the visual aid of exerted torque on an oscilloscope. The measured values were the means of two trials.

**Figure 1:**
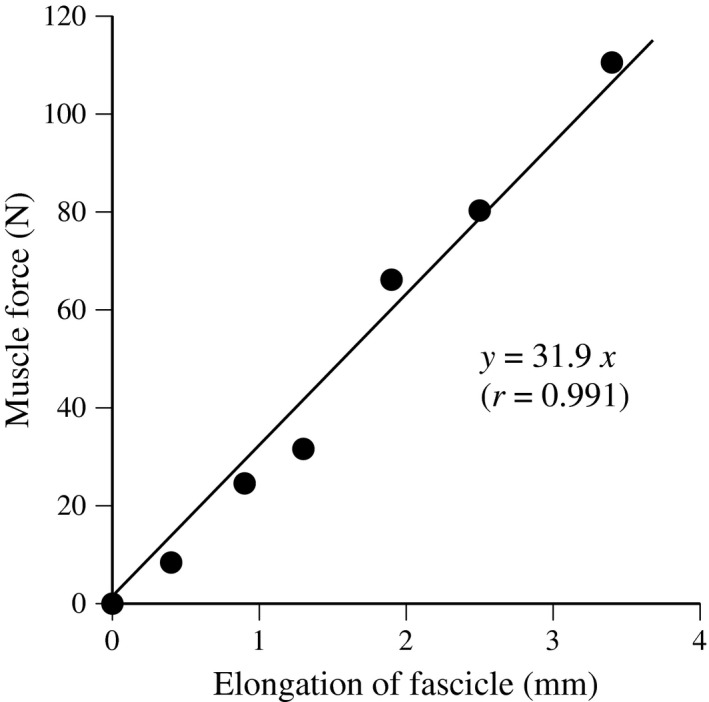
Typical example of muscle force and fascicle length during the measurement of active muscle stiffness.

A real‐time ultrasonic apparatus was used to obtain a longitudinal ultrasonic image of MG to measure fascicle length during fast stretching. Ultrasonic images were stored at 98 Hz in the computer memory of the apparatus (Kubo 2014; Kubo et al. 2015). An electric signal was superimposed on the images to synchronize them to the torque, joint angle, and electromyographic activity. The location of the probe, analysis of fascicle length, and calculated muscle force were similar to those for the measurement of passive muscle stiffness, as described above. The slope of muscle force–fascicle length curve between 10 deg and 2 deg was defined as active muscle stiffness (Fig. 1) (Kubo 2014; Kubo et al. 2015). In our previous study (Kubo 2014), the repeatability of active muscle stiffness measurements was investigated on two separate days with nine young males. The test–retest correlation coefficient (*r*) and the coefficient of variation were 0.870 and 10.4% for 30% MVC, 0.865 and 10.3% for 50% MVC, and 0.888 and 10.9% for 70% MVC.

### Stiffness and hysteresis of tendon structures

The posture of the subject (except for the ankle angle) and procedure used were similar to those for measurements of passive and active muscle stiffness, as described above. The ankle joint was set at 0 deg with the knee joint at full extension. Prior to the test, the subject performed a standardized warm‐up (e.g., static stretching) and submaximal contractions to become accustomed to the test procedure. In this study, the measurements of tendon properties were executed at two different speeds, that is, ramp and ballistic contractions. In ramp contractions, subjects were instructed to develop a gradually increasing force from a relaxed state to MVC within 5 sec, followed by gradual relaxation within 5 sec (e.g., Kubo et al. 2002, 2014). In ballistic contractions, subjects were instructed to contract as strongly and rapidly as possible, followed by sudden relaxation. The tasks for ramp and ballistic contractions were repeated twice per subject with at least 2 min between trials. Torque signals were analog‐to‐digital converted as a sampling rate of 1 kHz and analyzed by a computer.

A real‐time ultrasonic apparatus was used to obtain a longitudinal ultrasonic image of MG during the contraction. The ultrasonic images were recorded on a videotape at 60 Hz, synchronized with recordings of a clock timer for subsequent analyses. The displacement of the point at which one fascicle was attached to the aponeurosis was considered to indicate the lengthening of tendon structures. However, the tendon displacement is attributed to both angular rotation and contractile tension, since any angular joint rotation occurs in the direction of ankle plantar flexion during an “isometric” contraction. To monitor ankle joint angular rotation, an electrical goniometer (Penny and Giles) was placed on the lateral aspect of the ankle. To correct the measurements taken for the elongation of the tendon structures, additional measurements were made under passive conditions. Displacement of the point at which one fascicle was attached to the aponeurosis caused by rotating the ankle from 0 deg to 9 deg was digitized in sonographs taken as described above. Thus, for each subject, displacement of the point at which one fascicle was attached to the aponeurosis obtained from the ultrasound images could be corrected for that attributed to joint rotation alone (Magnusson et al. 2001; Kubo et al. 2014). In this study, only values corrected for angular rotation are reported.

**Figure 2:**
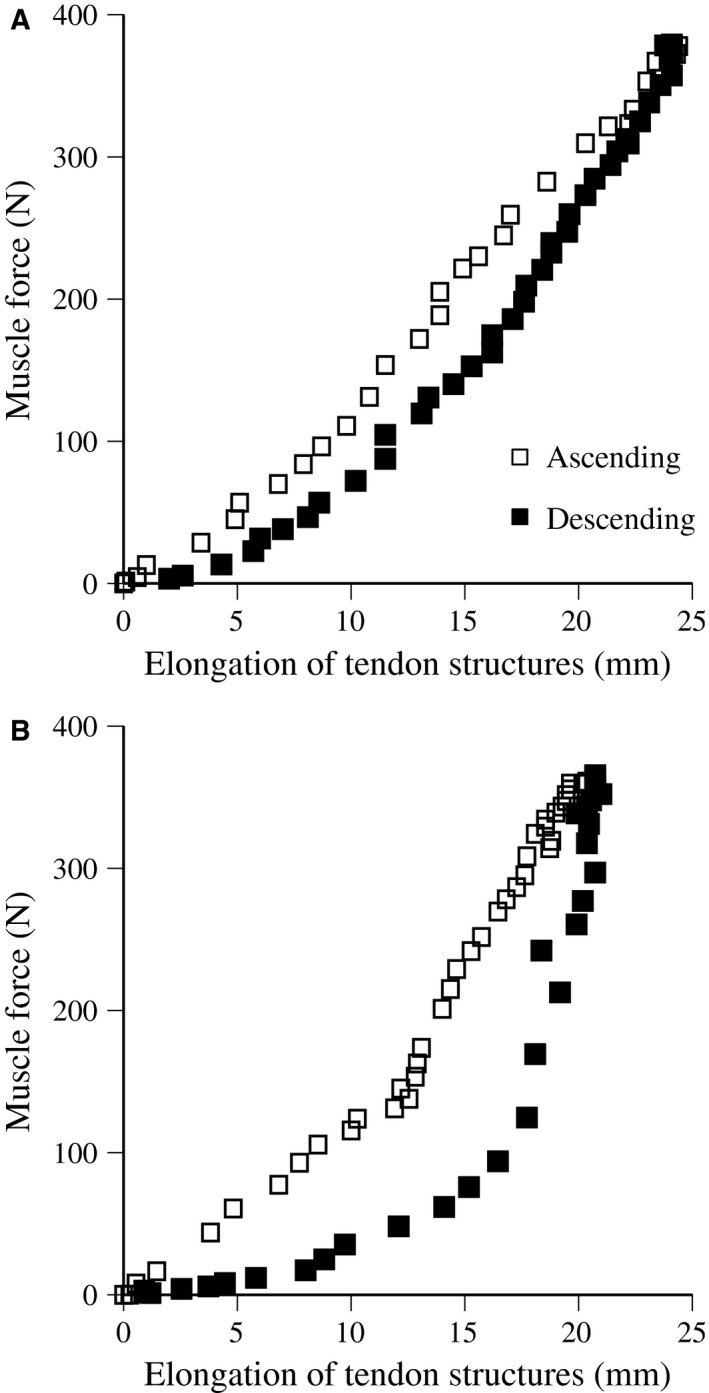
Typical example of muscle force and elongation of tendon structures during ramp (A) and ballistic (B) contractions.

Torque measured was converted to muscle force by the same procedure used to measure passive muscle stiffness. Figure 2 presented a typical example of the relationships between *F*_m_ and elongation of tendon structures during ramp and ballistic contractions. In this study, muscle force and elongation of tendon structures above 50% of MVC were fit to a linear regression equation, the slope of which was adopted as tendon stiffness (Kubo et al. 2002, 2014). The area within the force–elongation loop, as a percentage of the area beneath the curve during the ascending phase, was calculated as hysteresis (Kubo et al. 2002, 2014). In a preliminary study, the repeatability of tendon structures stiffness and hysteresis measurements was investigated on two separate days with 10 young males. The test–retest correlation coefficient (*r*) and the coefficient of variation for stiffness were 0.91 and 9.5% for ramp contractions and 0.87 and 9.0% for ballistic contractions. The test–retest correlation coefficient (*r*) and the coefficient of variation for hysteresis were 0.87 and 14.4% for ramp contractions and 0.83 and 12.9% for ballistic contractions.

### Jump performances and joint stiffness

Subjects performed three kinds of unilateral maximal jumps using only the ankle joint (no‐counter‐movement jump: noCMJ; counter‐movement jump: CMJ; drop jump: DJ) on the sledge apparatus. The load used was 50% of the body mass for each subject. Since the body mass of the subjects did not change after training, the load for each subject was the same before and after training. The vertical component of the ground reaction force (*F*_z_) was recorded from a force plate (Kistler, 9281B, Switzerland) attached to the force platform of the apparatus. Retroreflective marks were placed on the fifth metatarsophalangeal joint, lateral malleolus, and lateral epicondyle of the knee. During jumping, subjects were filmed with a digital high‐speed video camera at a sampling frequency of 250 Hz (VCC‐H1600C, Digimo, Tokyo, Japan).

Subjects had adequate practices (submaximal jumps to become accustomed to the test procedures) of these three jumps before testing. In all tests, they were instructed to jump to a maximal height. The test was repeated five times per subject, with at least 2 min between trials. In noCMJ, subjects initially kept the ankle position maximally dorsiflexed, and supported the load in this position. Subjects then started ankle movement until the ankle was fully plantar‐flexed and the toe lifted away from the force plate. In CMJ, subjects initially maintained the maximal plantar‐flexed position. Subjects then exerted plantar flexion torque to maximal dorsiflexion, and rebounded to start plantar flexion until the toe finally lifted away from the force plate. In DJ, the sliding table of this apparatus was moved to a height of 20 cm from the surface of the force plate to the sole of their foot with the assistance of an experimenter. Subjects were dropped down from a height of 20 cm. After landing on the edge of the force plate, they arrested the falling motion by eccentrically plantar flexing. They then started plantar flexion and took off. We excluded trials in which the knee joint was flexed slightly according to images taken by the high‐speed video camera. In our previous study (Kubo et al. 2007a) with five males, the optimal drop height for DJ was investigated. In the concrete, we determined the effects of the drop height (10, 20, 30, and 40 cm) on jump heights of DJ. As a result, the maximal jump heights of DJ were observed using the drop height of 20 cm. Furthermore, the coefficient of variation of the three measurements was 5.2% for noCMJ, 4.6% for CMJ, and 7.1% for DJ (Kubo et al. 2007a).

The ankle joint angle and jump height were measured using open‐source image analysis software (Image J, NIH, Bethesda, MD). Assuming that the displacement of the retroreflective landmark of the lateral malleolus was equal to that of the center of mass, jump height was defined as the maximum displacement of the retroreflective landmark of the lateral malleolus from the resting position (the ankle joint angle was 0 deg) (Kubo et al. 2007a,b). Three individual jump height recordings, excluding the largest and smallest values, were averaged.

Ankle joint torque (TQ) during DJ was estimated from the following equation (Kubo et al. 2007a,b):

$$\text{TQ}=F_{z}\cdot L_{1}\cdot \text{cos}\left(A_{J}\right)$$

where *F*_z_, *L*_1_, and *A*_J_ are the vertical component of the ground reaction force, the length from the estimated center of the ankle joint to the ball of the foot (measured for each subject), and the ankle joint angle, respectively. Ankle joint stiffness was calculated as a change in joint torque divided by the change in the ankle joint angle during the eccentric phase (Kubo et al. 2007a,b). In our previous study (Kubo et al. 2007a), the coefficient of variation of the three measurements for joint stiffness was 8.2%.

### Statistical analysis

Values are reported as means ± standard deviation. A two‐way analysis of variance (ANOVA) with repeated measures was used to detect significant effects of time (before and after training) and torque level (%MVC) on increments in torque and changes in fascicle length. Regarding the other variables, differences in time (before and after training) and mode (PLY and ISO) were tested using a two‐way ANOVA with repeated measures {2 (test times) × 2 (modes)}. The *F* ratios for main effects and interactions were considered to be significant. When ANOVA revealed significant main effects for mode and time, and whether a significant interaction existed between them, we returned to an one‐way ANOVA with repeated measures to detect any significant changes from before training. Statistical computations were performed using IBM SPSS Statistics (version 19). Significance was set at *P* < 0.05.

## Results

The muscle thickness of the plantar flexors significantly increased by 5.7 ± 2.6% with PLY and 5.5 ± 2.3% with ISO (both *P* < 0.001, Table 1). No significant difference in the relative increase in muscle thickness was found between PLY and ISO (*P* = 0.804). MVC significantly increased by 4.4 ± 5.0% with PLY (*P* = 0.013) and 22.1 ± 14.2% with ISO (*P* < 0.001) (Table 1). The relative increase in MVC was significantly greater with ISO than with PLY (*P* = 0.001).

**Table 1:** Morphological and mechanical properties of muscles for plyometric and isometric training protocols [mean (SD)]

	Plyometric training		Isometric training	
	Before	After	Before	After
MVC (Nm)	103.3 (7.6)	107.3 (9.4)*	104.3 (16.7)	124.8 (15.0)***
Muscle thickness of MG (mm)	17.7 (1.7)	18.8 (2.2)**	18.1 (1.8)	19.0 (1.9)***
Muscle thickness of LG (mm)	16.0 (2.3)	16.9 (2.4)**	15.5 (2.5)	16.4 (2.6)**
Muscle thickness of SOL (mm)	15.3 (2.5)	16.1 (2.4)***	15.9 (1.9)	16.8 (2.4)**
Muscle thickness of PF (mm)	16.3 (1.5)	17.3 (1.6)***	16.5 (1.7)	17.4 (1.9)***
Active muscle stiffness at 30%MVC (N·mm^−1^)	32.3 (8.6)	44.6 (9.6)**	35.9 (10.7)	40.3 (8.1)
Active muscle stiffness at 50%MVC (N·mm^−1^)	48.2 (15.5)	77.0 (24.3)**	49.4 (14.2)	60.4 (18.5)
Active muscle stiffness at 70%MVC (N·mm^−1^)	63.9 (17.8)	108.4 (16.7)***	72.4 (16.8)	89.5 (27.4)
Passive muscle stiffness (N·mm^−^)	7.35 (3.30)	7.30 (1.86)	7.39 (3.09)	7.47 (2.17)

Significantly different from before (*P < 0.05, **P < 0.01, ***P < 0.001). MVC, maximum voluntary contraction; MG, medial gastrocnemius muscle; LG, lateral gastrocnemius muscle; SOL, soleus muscle, PF, plantar flexor muscles.

**Figure 3:**
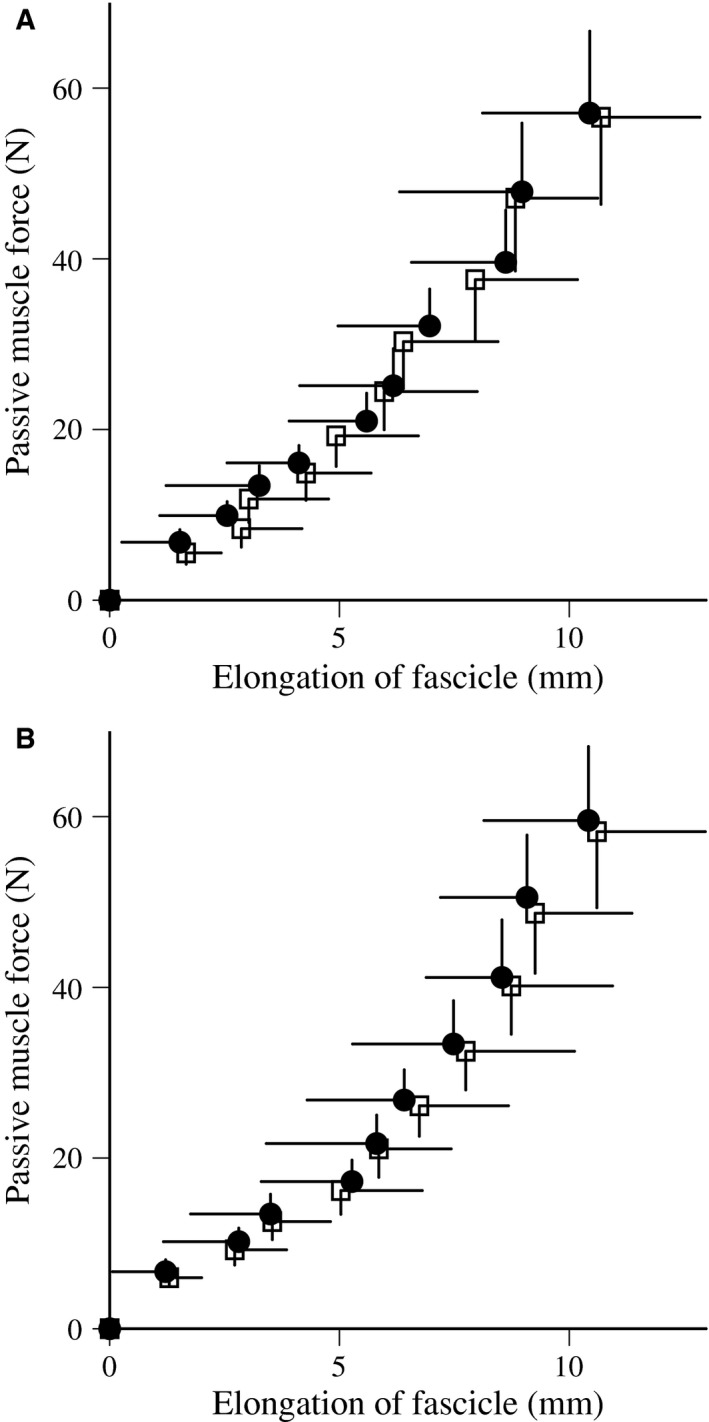
Relationships between passive muscle force and elongation of the fascicle during slow stretching before (open) and after (closed) plyometric (A) and isometric (B) training (mean ± standard deviation).

The relationships between passive muscle force and elongation of fascicles during slow stretching are shown in Figure 3. In PLY and ISO, passive muscle stiffness did not change after training (Table 1).

**Figure 4:**
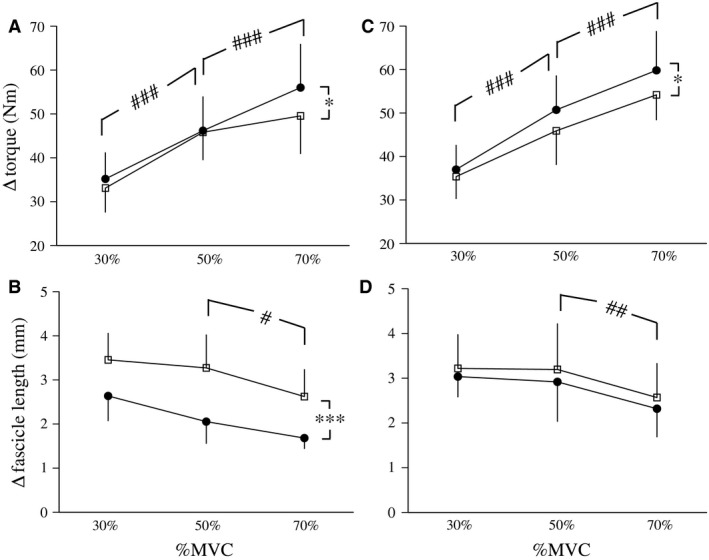
Changes in torque (A and C) and fascicle length (B and D) during fast stretching before (open) and after (closed) plyometric (A and B) and isometric (C and D) training (mean ± standard deviation). Significant difference from before: **P < 0.01, ***P < 0.001. Significant difference from the preceding torque level exerted: ^##^ *P* < 0.01, ^###^ *P* < 0.001.

Regarding increases in torque during fast stretching, the effects of time (*P* < 0.001 for PLY and ISO) and torque level (*P* = 0.044 for PLY, *P* = 0.012 for ISO) were significant, whereas the effect of the interaction between time and torque level was not (*P* = 0.083 for PLY, *P* = 0.402 for ISO) (Fig. 4A and C). Regarding changes in fascicle length during fast stretching, the effect of torque level (*P* < 0.001 for PLY and ISO) was significant, whereas that of the interaction between time and torque level was not (*P* = 0.437 for PLY, *P* = 0.971 for ISO) (Fig. 4B and D). Changes in fascicle length during fast stretching were significantly lower after training with PLY (*P* < 0.001), but not with ISO (*P* = 0.308) (Fig. 2B and D). Active muscle stiffness at all torque levels significantly increased after training with PLY, but not with ISO (*P* = 0.135 for 30%MVC, *P* = 0.359 for 50%MVC, and *P* = 0.214 for 70%MVC) (Table 1). Regarding PLY and ISO, no significant differences were observed in mEMGb/mEMGa ratio at any torque level between before and after training.

**Figure 5:**
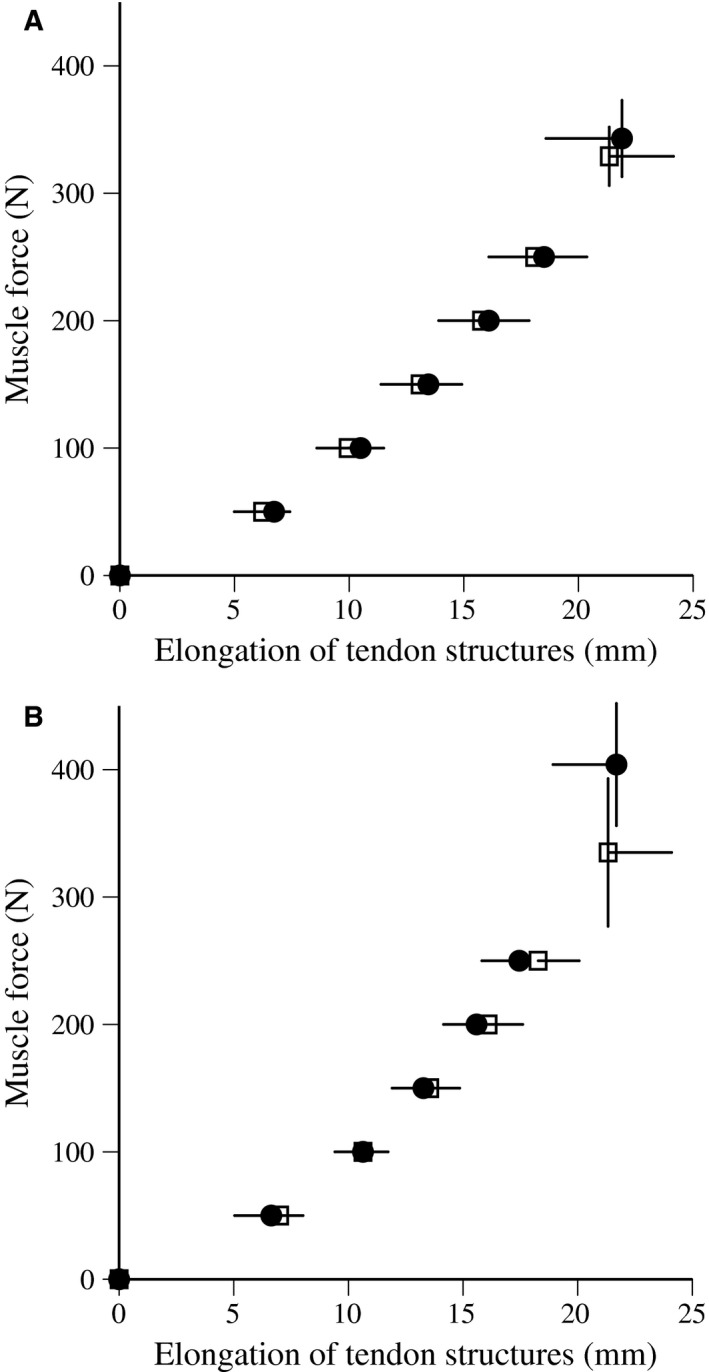
Relationships between estimated muscle force and elongation of tendon structures during ramp contractions before (open) and after (closed) plyometric (A) and isometric (B) training (mean ± standard deviation).

**Figure 6:**
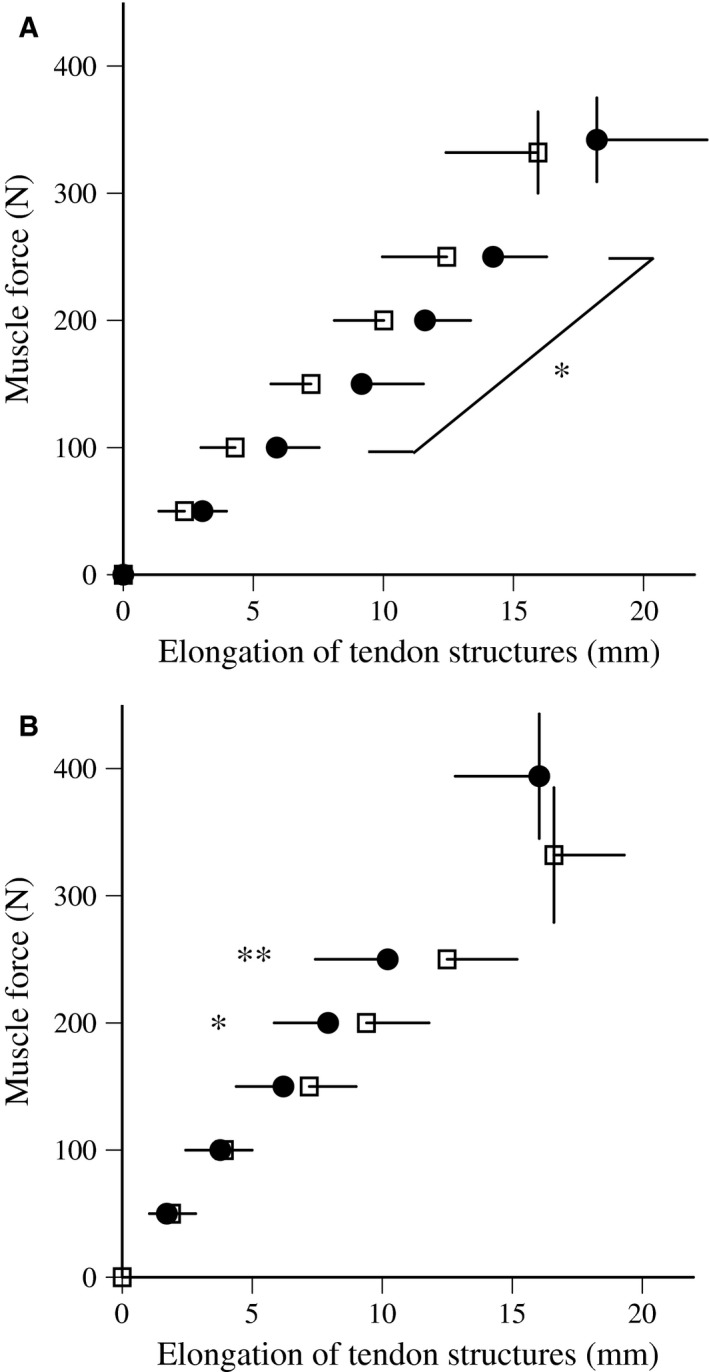
Relationships between estimated muscle force and elongation of tendon structures during ballistic contractions before (open) and after (closed) plyometric (A) and isometric (B) training (mean ± standard deviation). Significant difference from before: *P < 0.05, **P < 0.01, ***P < 0.001.

The relationships between muscle force and elongation of tendon structures during ramp contractions are shown in Figure 5. Neither protocol produced a significant difference in tendon elongation values at any force production level after training. The relationships between muscle force and elongation of tendon structures during ballistic contractions are shown in Figure 6. Tendon elongation values above 100 N were significantly longer after training with PLY, and maximal tendon elongation after training tended to be greater than that before (*P* = 0.063). On the other hand, tendon elongation values above 200 N were significantly shorter after training for ISO. Regarding ramp and ballistic contractions, the stiffness of tendon structures significantly increased after training with ISO (42.1 ± 48.2% for ramp, 27.3 ± 28.4% for ballistic), but not with PLY (Table 2). In ramp and ballistic contractions, hysteresis did not change after training with PLY and ISO. Furthermore, no significant change in the cross‐sectional area of tendons was found after training with PLY and ISO (Table 2).

**Table 2:** Morphological and mechanical properties of tendon structures for plyometric and isometric training protocols [mean (SD)]

		Plyometric training		Isometric training	
		Before	After	Before	After
Ramp	Maximal elongation (mm)	21.3 (2.8)	21.9 (3.3)	21.3 (2.8)	21.7 (2.8)
	Stiffness (N·mm^−1^)	22.6 (5.9)	23.3 (5.0)	23.7 (5.4)	32.5 (8.7)**
	Hysteresis (%)	20.5 (11.3)	18.5 (7.9)	20.4 (10.9)	18.6 (9.2)
Ballistic	Maximal elongation (mm)	15.9 (3.5)	18.2 (4.2)	16.6 (2.7)	16.0 (3.2)
	Stiffness (N·mm^−1^)	20.7 (5.9)	20.9 (5.8)	19.2 (4.0)	24.2 (6.5)**
	Hysteresis (%)	34.9 (8.9)	35.4 (10.1)	35.7 (9.3)	34.3 (10.0)
	Cross‐sectional area (mm^2^)	65.8 (7.8)	64.8 (7.1)	64.9 (7.8)	66.0 (7.8)

Significantly different from before (**P < 0.01).

Table 3 shows the variables measured during the three kinds of jumping tests before and after training. In PLY and ISO, no significant differences were observed in the ankle angle at the lowest position in any jumping test between before and after training. The angular velocities at the eccentric and concentric phases did not change after training, except for in the concentric phase of SJ for ISO. In PLY, jump heights significantly increased by 58.4 ± 32.9% for noCMJ, 50.2 ± 19.7% for CMJ, and 47.4 ± 35.9% for DJ (all *P* < 0.001). In ISO, jump height significantly increased by 20.9 ± 20.3% for SJ (*P* = 0.011), but not for CMJ (*P* = 0.088) or DJ (*P* = 0.171). Joint stiffness significantly increased by 31.0 ± 29.2% with PLY (*P* = 0.006), but not with ISO (*P* = 0.121).

**Table 3:** Ankle angle, angular velocity, performance, and joint stiffness during jumping tests for plyometric and isometric training protocols [mean (SD)]

		Plyometric training		Isometric training	
		Before	After	Before	After
Ankle angle at the lowest position (deg)	noCMJ	−24.0 (7.4)	−23.3 (5.3)	−23.7 (6.2)	−25.8 (4.4)
	CMJ	−23.6 (7.1)	−25.3 (8.0)	−24.7 (5.9)	−25.5 (3.4)
	DJ	−20.5 (7.8)	−19.3 (8.5)	−18.9 (8.2)	−20.8 (7.5)
Angular velocity during eccentric phase (deg·s^−1^)	CMJ	73.5 (13.2)	82.1 (15.2)	81.0 (15.7)	82.6 (13.0)
	DJ	153.4 (30.1)	145.0 (17.8)	167.1 (58.7)	153.0 (43.1)
Angular velocity during concentric phase (deg·s^−1^)	noCMJ	106.6 (24.9)	127.1 (23.8)	108.3 (20.9)	118.7 (23.0)*
	CMJ	122.5 (17.5)	132.7 (16.0)	134.2 (12.8)	138.7 (12.9)
	DJ	171.6 (52.3)	182.5 (45.8)	190.5 (50.0)	193.4 (41.4)
Jump height (cm)	noCMJ	16.4 (4.4)	25.0 (4.6)***	17.1 (4.5)	20.0 (3.3)*
	CMJ	19.1 (3.5)	28.3 (4.1)***	20.7 (5.1)	23.3 (3.0)
	DJ	19.4 (5.4)	27.1 (4.5)***	19.6 (4.9)	21.7 (3.7)
Joint stiffness (Nm·deg^−1^)	DJ	6.52 (2.00)	8.28 (2.06)**	6.98 (2.44)	7.80 (2.75)

Significantly different from before (*P < 0.05, **P < 0.01, ***P < 0.001). noCMJ, no‐counter‐movement jump; CMJ, counter‐movement jump; DJ, drop jump.

## Discussion

The most important result of this study is that the extensibility of tendon structures during ballistic contractions and active muscle stiffness during fast stretching significantly increased after plyometric training, but not for isometric training. To the best of our knowledge, this is the first study to demonstrate training‐induced changes in tendon properties during ballistic contractions and muscle stiffness under active conditions.

So far, there are conflicting findings on the effects of plyometric training on tendon properties during ramp contractions (Burgess et al. 2007; Kubo et al. 2007b; Foure et al. 2009, 2010b; Wu et al. 2010). A novel result of this study is that extensibility during ballistic contractions increased after PLY (Fig. 6A). Before training, the elongation of tendon structures at a lower torque level (i.e., toe region) was lower during ballistic contractions than during ramp contractions, whereas no significant difference was observed in the slope at linear region (i.e., stiffness). Pioletti et al. (1999) also reported that differences between stress–strain curves of the anterior cruciate ligament obtained at different strain rates were mainly observed in the toe region. They stated that more water moved through the collagen fibers during the early arrangement of the fibers. Therefore, it is possible that the water content within the tendons decreases after plyometric training. Furthermore, Hirayama et al. (2012) showed that the magnitude of tendon elongation during counter‐movement jump increased after a single practice session. This finding indicates that acute changes in tendon elongation during jumping were repeated and accumulated during the experimental period (12 weeks). In any case, it can be said that the results obtained on tendon properties for PLY are desirable for storing elastic energy during stretch‐shortening cycle exercises. Furthermore, we can observe this new finding through the measurement of tendon properties during ballistic contractions.

In this study, relative increases in tendon stiffness by ISO were approximately 35% for ramp and ballistic contractions, and were slightly lower than previous findings using isometric training. Previous studies demonstrated that tendon stiffness increased by more than 50% after isometric training (e.g., Burgess et al. 2007; Kubo et al. 2012). It is difficult to compare the degree of increases in tendon stiffness by isometric training due to differences in the respective experimental conditions among these studies. Kubo et al. (2012) used the same experimental conditions on the measured site (plantar flexor muscles), intensity and duration during contractions (80% of MVC and 15 sec), and training period (12 weeks) as this study, and the relative increase in tendon stiffness was 50.3% after training. However, the amount of training in this study (10 sets, three times per week) was lower than that in Kubo et al. (2012) (15 sets, four times per week). Therefore, the discrepancy described in the relative increase in tendon stiffness may be related to difference in the amount of training during the experimental period.

To date, few studies have attempted to investigate training‐induced changes in tendon hysteresis (Kubo et al. 2002; Reeves et al. 2003; Foure et al. 2010b). Foure et al. (2010b) reported that tendon hysteresis significantly decreased by 35% after 14 weeks of plyometric training. The present results on tendon hysteresis differed from the finding of Foure et al. (2010b). On the other hand, a more recent study showed that the shortening velocity of tendons in the initial half of the concentric phase during drop jumps significantly increased after 12 weeks of plyometric training (Hirayama et al. 2017). This finding implies that tendon hysteresis decreases after plyometric training. In this study, however, hysteresis of tendon structures for ramp and ballistic contractions did not change after PLY (Table 2). The shortening velocity of tendon structures during the descending phase of ballistic contractions was approximately 35 mm·sec^−1^, which was markedly lower than that during jumping (60–200 mm·sec^−1^; Hirayama et al. 2017; Ishikawa et al. 2005; Kurokawa et al. 2003). In future studies, we need to investigate training‐induced changes in tendon hysteresis during higher shortening conditions than ballistic contractions in this study.

In PLY and ISO, passive muscle stiffness did not change after training. Previous studies showed that passive muscle stiffness (or passive tension) was related to the mechanical properties of connective tissue elements in parallel with the muscle belly (Jewell and Wilkie 1958; Gajdosik 2001) and titin isoforms (Wang et al. 1991; Spierts et al. 1997). Ducomps et al. (2003) demonstrated using animals that the collagen concentration and passive stiffness of muscles significantly increased after jump training. Unfortunately, we have no definite information on changes in the collagen concentrations of the connective tissue elements of muscle. To the best of our knowledge, there have been no studies to show increases in collagen concentrations in human muscles after several months of training in vivo. Furthermore, previous studies reported that the titin isoforms of human muscles did not change after plyometric training (McGuigan et al. 2003; Pellengrino et al. 2016). Collectively, these findings and the present results on passive muscle stiffness indicate that the collagen concentrations and titin isoforms of muscles were not changed enough by PLY and ISO to enhance passive torque.

Another interesting result of this study is that active muscle stiffness increased with PLY, but not with ISO. The influence of the stretch reflex on the measurement of active muscle stiffness needs to be considered when muscle stiffness is assessed under active conditions. According to previous findings (Voigt et al. 1998; Potach et al. 2009), the excitability of the short‐latency stretch reflex of muscles did not change after plyometric training. In this study, changes in torque and fascicle length were analyzed during a 60‐msec period after the stretch, since this time period was selected in order to avoid any potential neural effects (Blanpied and Smidt 1992; Kubo 2014). In addition, no significant differences in the mEMGb/mEMGa ratio between before and after training were found for PLY or ISO. Therefore, we may say that enhancements in active muscle stiffness after plyometric training did not relate to changes in the excitability of the short‐latency stretch reflex.

As an other possible reason for the enhancement observed active muscle stiffness with PLY, we suggest training‐induced changes in the mechanical properties of cross‐bridges and actin, myosin, and titin filaments within the sarcomere. Unfortunately, we cannot discuss this point using the results obtained in this study. In any case, the results shown in Figure 4 indicate that increases in active muscle stiffness with PLY were caused by decreases in fascicle lengthening, not increases in torque, during the short range stretch experiment. This result implies that the mechanical properties of cross‐bridges and filaments within the sarcomere become less extensible by repetitive stresses during 12 weeks of PLY. On the other hand, it is possible that decreases in fascicle lengthening during stretching are related to an increase in the extensibility of tendon structures during ballistic contractions. Regardless, further studies using isolated animal muscles are needed in order to clarify this point.

In this study, joint stiffness significantly increased with PLY, but not with ISO (Table 3). This result is consistent with previous findings (Spurrs et al. 2003; Kubo et al. 2007b). Furthermore, no significant differences were observed in the changes induced by plyometric training in muscle activities during jumping in our previous study (Kubo et al. 2007b). The present results imply that the increase noted in joint stiffness after plyometric training is related to that in active muscle stiffness, because of the absence of changes in passive muscle stiffness and tendon stiffness. Unfortunately, there was no significant correlation coefficient between relative increases in joint stiffness and active muscle stiffness at any torque level for PLY (data not shown). The duration (200 ± 37 msec) of the eccentric phase during DJ for calculating joint stiffness was markedly longer than that for calculating active muscle stiffness (60 msec). Therefore, joint stiffness may be affected by the long‐latency stretch reflex. Potach et al. (2009) stated that the long‐latency stretch reflex may be altered by plyometric training, whereas the latency time of the short‐latency stretch reflex did not change. We are currently considering the development of a method to assess active muscle stiffness including the stretch reflex (in particular the long‐latency stretch reflex).

In this study, we need to discuss the limitations and assumptions of the methodology followed. We defined the relative contribution of MG to force production during plantar flexion as the percentage of the physiological cross‐sectional area of this muscle to that of the plantar flexor muscles, as described in Fukunaga et al. (1996). In this study, no significant differences were observed in the relative increase in muscle thickness among MG, LG, and SOL (Table 1). This result implies that the relative contribution of each constituent to torque production is similar before and after training. We also used the estimated moment arm length by taking the difference in limb length between subjects into account based on the finding of Grieve et al. (1978). Sugisaki et al. (2015) more recently reported that moment arm length increased by 5.5% (approximately 1.1 mm) after 12 weeks of resistance training, and, thus, they stated that the impact of increases in moment arm length may be small or even negligible. Therefore, we considered the muscle force calculation based on the assumptions described above to be valid to study changes in muscle and tendon properties after training.

Furthermore, we investigated the mechanical properties of tendon structures (including outer tendon and aponeurosis), but not those of outer tendon (i.e., Achilles tendon), in this study. Previous studies demonstrated that there was difference in the mechanical properties between outer tendon and aponeurosis (e.g., Magnusson et al. 2003). We considered that the dynamics of muscle fibers and muscle function would be more closely related to the mechanical properties of tendon structures (including outer tendon and aponeurosis) than those of outer tendon, although this point was not proved experimentally so far.

In conclusion, joint stiffness and active muscle stiffness increased after plyometric training, but not after isometric training. Furthermore, the extensibility of tendon structures during ballistic contractions was enhanced after plyometric training, and the stiffness of tendon structures increased after isometric training. These results imply that changes in muscle and tendon properties after plyometric training favor performance during stretch‐shortening cycle exercises.

## Conflict of Interest

None declared.
